# Exposure, Postexposure, and Density-Mediated Effects of Atrazine on Amphibians: Breaking Down Net Effects into Their Parts

**DOI:** 10.1289/ehp.8405

**Published:** 2005-09-07

**Authors:** Jason R. Rohr, Tyler Sager, Timothy M. Sesterhenn, Brent D. Palmer

**Affiliations:** 1 Penn State Institutes of the Environment, and; 2 Department of Entomology, Penn State University, University Park, Pennsylvania, USA; 3 Department of Biology, University of Kentucky, Lexington, Kentucky, USA

**Keywords:** amphibian declines, atrazine, density dependence, development, endocrine disruption, nonlinear dose response, pesticide, postexposure effects, salamander

## Abstract

Most toxicology studies focus on effects of contaminants during exposure. This is disconcerting because subsequent survival may be affected. For instance, contaminant-induced mortality can be later ameliorated by reduced competition among the survivors, a concept we refer to as “density-mediated compensation.” Alternatively, it can be exacerbated by toxicant effects that persist or appear after exposure, a phenomenon we term “carryover effects.” We developed a laboratory framework for testing the contribution of exposure, density-mediated, and carryover effects to net survival, by exposing embryos and larvae of the streamside salamander (*Ambystoma barbouri*) to atrazine (0, 4, 40, 400 ppb; 3 ppb is the U.S. drinking water maximum) and quantifying survival during and 14 months after exposure. Atrazine is the most commonly used herbicide in the United States and a documented endocrine disruptor. We show that atrazine-induced mortality during exposure was ameliorated by density-dependent survival after exposure, but complete density-mediated compensation was precluded by significant carryover effects of atrazine. Consequently, salamanders exposed to ≥4 ppb of atrazine had significantly lower survival than did control animals 14 months postexposure. The greatest change in survival occurred at low exposure concentrations. These nonlinear, long-term, postexposure effects of atrazine have similarities to effects of early development exposure to other endocrine disruptors. Together with evidence of low levels of atrazine impairing amphibian gonadal development, the results here raise concerns about the role of atrazine in amphibian declines and highlight the importance of considering persistent, postexposure effects when evaluating the impact of xenobiotics on environmental health.

A standard approach for evaluating the threat that a xenobiotic poses to environmental health is to test whether environmentally realistic concentrations have adverse effects on organisms that may be harbingers of possible risk to humans or ecosystem services. Amphibians have long been considered one of these bioindicators of environmental stress, especially for xenobiotics, because many amphibians experience aquatic and terrestrial stressors, play vital roles in communities, are sensitive to contaminants, complete their life cycles near fields where pesticides are applied, and have vulnerable embryo and larval stages whose development coincides with pesticide applications (Blaustein and Kiesecker 2002; Hayes et al. 2003; Rohr and Madison 2003; Rohr et al. 2004; Westerman et al. 2003). Most toxicologic studies, however, examine the effects of xenobiotics only during exposure (Figure 1) (Jensen and Forbes 2001; Ng and Keough 2003), and amphibian studies certainly are not an exception (Rohr and Palmer 2005). This is a concern because the effects of a contaminant can be unchanged, exacerbated, or ameliorated once contaminant exposure has ceased. For instance, if a stressor, such as a toxicant, induces juvenile mortality, the survivors may experience substantially less mortality than controls after exposure because of reduced competition for resources (Figure 1) (e.g., Moe et al. 2001). We refer to this concept as density-mediated compensation, a concept with a long history of study in the ecologic and wildlife literature. Alternatively, the effects of a pollutant may persist or appear after exposure (e.g., Hernandez-Avila et al. 1996; Quistad et al. 2003; Rice 1996), a phenomenon we refer to as carryover effects of the contaminant (Figure 1).

Ideally, decisions regarding environmental health should be made with an understanding of the effects that xenobiotics have on population growth rates (Forbes and Calow 2002; Schmidt 2004); however, this can be challenging for amphibians because they have relatively long generation times (often ≥ 3 years) and are frequently not amenable to life-cycle completion under tractable conditions. Moreover, the sheer number of registered chemicals (tens of thousands) and species to be tested and the complexities of population growth rate analyses may make this approach too time-consuming considering the urgent need to identify causal factors in the global decline of amphibians (Houlahan et al. 2000; Stuart et al. 2004). Researchers have suggested that a more practical and efficient alternative may be to study the effects of contaminants across life stages using amphibians reared under semi-natural conditions where postexposure effects and density-dependent regulation are permitted (Rohr et al. 2004; Sih et al. 2004). The proxy for population-level effects would be the net effect of the contaminant at some point near reproductive age, where the net effect is defined as the sum of exposure effects, carryover effects, and density-mediated compensation (Figure 1). We used this approach to investigate the net effects of the herbicide atrazine (0, 4, 40, and 400 ppb) on the streamside salamander, *Ambystoma barbouri.*

We focused on atrazine for several reasons. Atrazine is the most widely used pesticide in the United States, and possibly the world, and is one of the most common contaminants in groundwaters and surface waters [U.S. Department of Agriculture (USDA) 2002; U.S. Environmental Protection Agency (U.S. EPA) 1994]. Thus, many amphibians are likely exposed to atrazine. Atrazine concentrations seldom exceed 20 ppb for long but are likely to remain low (parts per billion) for extended time periods, especially considering that reported half-lives in fresh water exceed 100 days (de Noyelles et al. 1980, 1989; Klaassen and Kadoum 1979). Near agricultural areas, atrazine levels have been reported as high as 500 ppb in ponds (de Noyelles et al. 1982), 200 ppb in streams (U.S. Geological Survey 2000), and 40 ppb in rain (Nations and Hallberg 1992).

Recent studies have raised concerns regarding the effects of atrazine on amphibian fitness. The endocrine-disrupting properties of atrazine have been implicated in exposures between 25 and 0.1 ppb inducing gonadal abnormalities and hermaphroditism in leopard frogs (*Rana pipiens*) and African clawed frogs (*Xenopus laevis*) (Carr et al. 2003; Hayes et al. 2002a, 2002b, 2003; Tavera-Mendoza et al. 2002a, 2002b). Exposure to 3 ppb has increased tadpole mortality (Storrs and Kiesecker 2004) and susceptibility to infection (Kiesecker 2002).

The level of acute toxicity of a compound may not be as important as the persistence of its toxic effects (Rohr and Palmer 2005). Embryo and larval salamanders exposed to environmentally realistic atrazine concentrations have been reported to have an increased desiccation risk 8 months after exposure had ceased (Rohr and Palmer 2005), indicating that the carryover effects of atrazine on amphibian survival should be explored. The purpose of the present study was to determine the contributions of exposure, carryover, and density-mediated effects to the net survival of streamside salamanders 14 months after atrazine exposure.

## Materials and Methods

Postmetamorphic *A. barbouri* used in this study are from a static renewal experiment conducted by Rohr et al. (2004). Thirty-eight *A. barbouri* egg clutches were collected from Fossil Creek (Jessamine County, KY) in February 2003 and were mixed to homogenize genetic variation. Forty embryos were randomly placed in each of 48 aquaria containing 9.5 L of constantly aerated, charcoal-filtered, dechlorinated municipal tap water. Aquaria were maintained at 15°C on 12:12-hr photo-period, and their water was changed weekly. On day 16 of the experiment, after 99% of the embryos had hatched, we arbitrarily removed larvae from the aquaria to ensure that all had 24 larvae. This was done to obtain tissue samples for another study, to lower larval densities, and to reduce the starting variation in the number of larvae among aquaria.

We employed a 4 × 2 ×2 complete factorial design in which each aquarium was randomly assigned one of four ecologically relevant atrazine concentrations (0, 4, 40, or 400 ppb; 98% pure; Chemservice, West Chester, PA), one of two food-level treatments (low or high), and one of two hydroperiod treatments (presence or absence of a water level reduction). Atrazine was predissolved in acetone; all treatments contained 0.004% acetone, and concentrations were confirmed by flame ionization detection gas chromatography. Similar solvent concentrations were shown to have no effect on *A. barbouri* behavior, life history, or survival (Rohr et al. 2003a), and so a negative control was not included in this experiment. Low-food aquaria received 2.24 g (± 0.005 g) of live black worms (*Lumbriculus variegates*) twice a week, and larvae in the high-food aquaria were fed black worms *ad libitum*. In half the aquaria, the volume of water was incrementally reduced during water changes to simulate summer drying of water bodies, but atrazine concentration remained the same. Water volume did not drop below 2 cm. Mortality was quantified every other day throughout the exposure period, and animals were exposed to treatments until metamorphosis (mean ± SD, 59.9 ± 4.52 days; range, 47–70 days; for effects on embryos, larval growth, behavior, and timing of metamorphosis, see Rohr et al. 2004). In summary, there were three replicate aquaria of each treatment combination and a total of 12 aquaria exposed to each of the four atrazine concentrations.

After metamorphosis, *A. barbouri* from each aquarium were placed into terraria (18.5 cm in diameter, 7.5 cm high) so that the 48 aquarium replicates were preserved. Thus, density at metamorphosis carried over into the terrestrial stage, simulating what occurs in nature and allowing us to quantify any density-mediated compensation. Each terrarium contained approximately 6 cm of homogeneously mixed, organic top soil. Every week, we fed the salamanders vitamin- and mineral-enriched crickets *ad libitum* and added water to the terraria (if needed) to ensure that each had sufficient moisture. Terraria were maintained at room temperature on a 12:12-hr photoperiod. We recorded survival on 13 July 2004, a mean of 421 days (SD = ± 4.52; range, 410–433 days) since metamorphosis and last atrazine exposure. Survival was not recorded between metamorphosis and 13 July 2004 because postmetamorphic *A. barbouri* burrow into the substrate and digging them out greatly disturbs both the salamanders and the terraria. When possible, salamanders were treated humanely and with regard for alleviation of suffering.

**Figure 1:**
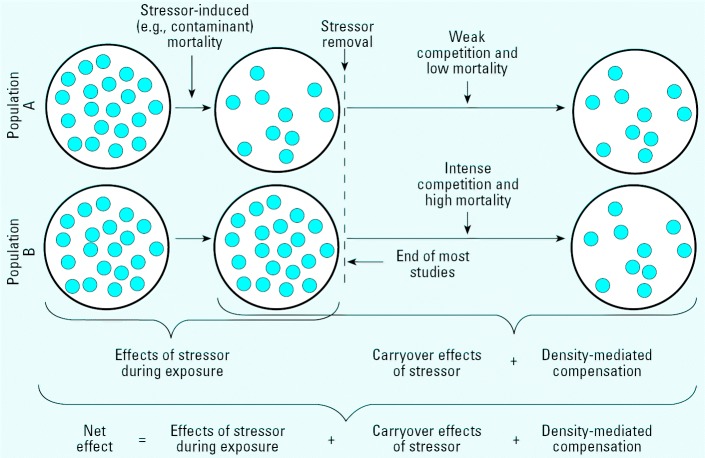
Heuristic model for the contribution of exposure, carryover, and density-mediated effects of a stres-sor to a stressor’s net effect on survival.

We used the general linear model to test for the main and interaction effects of atrazine (continuous predictor and log_10_-transformed), food level, and dry down on angularly transformed percent survival at metamorphosis (during atrazine exposure), after metamorphosis (carryover effect plus density-mediated compensation), and on 13 July 2004 (net atrazine effect; Figure 1). To test for carryover effects, we controlled for starting density in the postexposure phase (by incorporating survival to metamorphosis as a covariate in the statistical model) when examining postexposure survival. Because density-mediated compensation is driven by the different starting densities in the postexposure period, controlling for starting density removes density-mediated compensation, leaving any carryover effects of the contaminant (plus error; Figure 1). Where the main effect of atrazine was significant, we examined all pairwise comparisons among concentrations using Fisher’s least significant difference (LSD) tests.

To compare the magnitude (slope of regression) of atrazine’s exposure, post-exposure, carryover, and net effects, we examined all pairwise comparisons of these four effects using the general linear model, treating pairs of effects as repeated measures variables (i.e., we compared these effects within aquaria/terraria). An interaction between effect type and atrazine would indicate that mortality associated with atrazine depended upon the type of effect considered. The α-value for these tests (α = 0.0083) was modified for the number of comparisons using a Bonferroni adjustment. Comparisons with the carryover effect were conducted using the standardized residuals of survival at metamorphosis regressed against survival after metamorphosis.

The mean and variance of survival during the exposure and postexposure periods differed substantially (because of their different durations), making it difficult to visually evaluate the magnitude of atrazine effects across life stages. To facilitate visually comparing the exposure, postexposure, carryover, and net effects of atrazine, we standardized these effects using *Z*-scores so that the overall mean and SD in each phase were 0 and 1, respectively. Standardization does not affect statistical results.

## Results

The effect of atrazine on survival was independent of food level and dry down treatments for exposure, postexposure, carryover, and net effects (interactions including atrazine: *p* > 0.11). Thus, in this article, we focus strictly on the main effects of atrazine.

**Figure 2:**
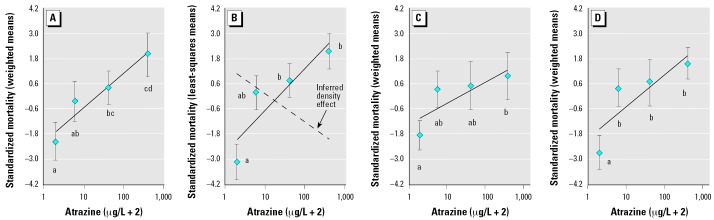
Effects of embryo and larval *A. barbouri* exposure to atrazine on survival through development, where the net effect 421 days after atrazine exposure (*D*) is broken into exposure (*A*), carryover (*B*), and carryover plus density-mediated effects (*C*). The exposure effect was the effect before metamorphosis; the carryover and density-mediated effects occurred after metamorphosis, and the net effect is the effect across both life stages. In (*A*), (*C*), and (*D*), values are the standardized weighted means. In (*B*), values are the standardized least-squares means, which are the weighted means adjusted for starting postmetamorphic density, a covariate incorporated into the statistical model. Standardizing means (zero mean and unit variance) facilitates comparing the effect of atrazine across life stages (see text for details). Error bars indicate SEs, and solid lines are best-fit lines; *n* = 12 for each treatment group. *p*-Values for the relationship between atrazine concentration and mortality are as follows: (*A*), *p* < 0.001; (*B*), *p* < 0.002; (C), *p* < 0.067; (*D*), *p* < 0.005. Different lowercase letters within each panel reflect significant differences (*p* < 0.05) among atrazine concentrations according to a Fisher’s LSD multiple comparison test. In (*B*), the effect of density-mediated compensation was inferred by subtracting the best-fit line for the carryover effect (*B*) from the best-fit line for the carryover effect plus density-mediated compensation (*C*).

During the exposure period, atrazine concentration was positively associated with mortality (*F*_1,40_ = 13.80, *p* < 0.001), with survival in control aquaria differing from that in aquaria containing 40 ppb (LSD, *p* = 0.027) and 400 ppb atrazine (LSD, *p* < 0.001; 0 vs. 4 ppb, *p* = 0.094; Figure 2A). Exposure concentration was also positively associated with mortality for the carryover effect (*F*_1,39_ = 10.71, *p* = 0.002). Survival of salamanders not exposed to atrazine was, once again, greater than the survival of animals exposed to 40 ppb (LSD, *p* = 0.039) and 400 ppb (LSD, *p* = 0.015; 0 vs. 4 ppb, *p* = 0.056; Figure 2B). The carryover effect analysis revealed that starting density in the postexposure phase was positively associated with postexposure mortality (*F*_1,39_ = 8.76, *p* = 0.005), indicating that the heavy mortality imposed by atrazine during the exposure period was ameliorated after exposure by density-mediated compensation (Figure 2B). Although the carryover effect was greater than density-mediated compensation, this compensation was substantial enough to offset a sizable portion of the carryover effect, resulting in a nonsignificant relationship between exposure concentration and net postexposure survival (*F*_1,40_ = 3.55, *p* = 0.067; Figure 2C).

Although density-mediated compensation neutralized the bulk of the atrazine-related carryover effect, it was not substantial enough to fully counteract the mortality associated with both the exposure and carryover effects. Consequently, exposure concentration was related positively to the net mortality across both the exposure and postexposure stages or mortality at the end of the study (*F*_1,40_ = 8.91, *p* = 0.005; Figure 2D). *A. barbouri* that were not exposed to atrazine had greater net survival than did *A. barbouri* exposed to 4 ppb (LSD, *p* < 0.013), 40 ppb (LSD, *p* < 0.007), or 400 ppb atrazine (LSD, *p* < 0.001; Figure 2D).

Pairwise comparisons of exposure, post-exposure, carryover, and net effects of atrazine on *A. barbouri* mortality revealed that the post-exposure effect (density-mediated + carryover effects) was significantly weaker than both the carryover (*F*_1,40_ = 17.58, *p* < 0.001) and net effects (*F*_1,40_ = 15.47, *p* < 0.001; Figure 2). No other comparisons were significant (*p* > 0.255).

## Discussion

We have shown that larval streamside salamander mortality attributed to atrazine exposure can be ameliorated by density-dependent processes after exposure, but that complete density-mediated compensation may be precluded by the long-term, postexposure effects of atrazine. Consequently, relative to control animals, *A. barbouri* previously exposed to concentrations of atrazine as low as 4 ppb had significantly lower survival 421 days after exposure, a result that was apparent only when considering the accumulation of both exposure and carryover effects. Atrazine at 4 ppb is only 1 ppb greater than the maximum allowable level in U.S. drinking water (U.S. EPA 2002) and a concentration to which amphibians may be chronically exposed (Hayes et al. 2003). In the process of measuring the effects of atrazine on *A. barbouri* survival, we developed a laboratory framework for quantifying exposure, carryover, density-mediated, and net effects of stressors on survival that may serve as a more practical alternative to the rigorous demands and complexities of population growth rate analyses.

Although we do not know when the post-exposure mortality occurred, we saw no evidence that there was substantial mortality soon after the salamanders were transferred to their terraria. Further, in a separate study on this species with similar exposure and rearing conditions, atrazine had no significant effect on larval survival, yet exposure was associated with hyperactivity and increased desiccation risk 8 months after exposure, with no detectable recovery from atrazine exposure (Rohr and Palmer 2005). These data suggest that exposure to atrazine early in development may have permanent effects on these salamanders. This conclusion is supported by our finding that the effect of atrazine during exposure did not differ from the magnitude of its carryover effect, once again suggesting that there was no recovery from atrazine exposure. Atrazine has been shown to disrupt neuroendocrine processes in amphibians (Hayes et al. 2002b, 2003; Larson et al. 1998), and this certainly is a possible mechanism for the observed long-term effects on behavior and survival.

Various studies have reported nonlinear relationships between atrazine concentration and amphibian responses, and many of these relationships have been nonmonotonic, with the largest change in response occurring at low exposure levels (Hayes et al. 2002b, 2003; Larson et al. 1998; Storrs and Kiesecker 2004). Endocrine disruptors commonly induce non-monotonic dose–response curves (Welshons et al. 2003), and thus the endocrine-disrupting potential of atrazine has been suggested as the cause of the documented nontraditional non-monotonic dose responses (Hayes et al. 2002b, 2003; Larson et al. 1998; Storrs and Kiesecker 2004). The relationship between atrazine concentration and survival in this study was nonlinear (logarithmic) but monotonic. However, we cannot rule out the possibility of a non-monotonic relationship because detection depends upon selecting the right concentrations to reveal any nonmonotonicity. The responses to atrazine recorded in *A. barbouri* have many consistencies with endocrine disruption. The largest adverse change in survival occurred at low exposure concentrations. Further, exposure to endocrine-disrupting compounds during critical developmental stages often induce irreversible effects (Bigsby et al. 1999), not unlike the long-term, postexposure effects observed here and in a previous study (Rohr and Palmer 2005). Virtually every response to atrazine we have quantified in this species using these concentrations has had the greatest response change at low concentrations (Rohr et al. 2004; Rohr and Palmer 2005; Rohr et al. unpublished data), suggesting that *A. barbouri* may be sensitive to marginal atrazine inputs into aquatic systems.

The deleterious, long-term effects of larval atrazine exposure suggest that encounters with contaminants after metamorphosis may not be necessary for contaminants to harm postmetamorphic amphibians, a life stage that often disproportionately affects population dynamics (Biek et al. 2002, Schmidt et al. 2005, Vonesh and De la Cruz 2002). This is important because exposure to substantial concentrations of contaminants is probably more likely before metamorphosis, because most amphibian embryos and larvae are strictly aquatic and cannot readily escape water bodies where many contaminants accumulate and concentrate.

Although the data in this laboratory study suggest that atrazine may have adverse effects on *A. barbouri*, we caution against extending this interpretation to populations in nature or to other amphibian species for the following reasons: *a*) causes of postmetamorphic mortality in the laboratory may not match those in the field; *b*) there is documented annual variation in the effects of atrazine on amphibians (Rohr et al. 2004); *c*) the strength of density-dependent processes may be different in the wild and for other species (Hellriegel 2000); *d*) species can differ in their susceptibility to contaminants (Bridges and Semlitsch 2000; Forbes and Calow 2002); and *e*) effects of contaminants can depend upon community structure (Relyea and Mills 2001; Rohr and Crumrine 2005), which was not incorporated into this study. Further, density-mediated compensation can be delayed by a generation or more; this, however, seems unlikely for atrazine because it is applied as a preemergent and thus enters ponds and wetlands in agriculture landscapes annually at relatively consistent times and quantities (Hayes et al. 2003, Huber 1993, Solomon et al. 1996). Consequently, larvae of many amphibian species are likely to be exposed to atrazine each spring, reducing the chances of cross-generational compensation. Nevertheless, the possibility of cross-generational compensation suggests that combining experiments that span life stages with demographic models might be particularly insightful for evaluating the population-level effects of environmental stress. Despite the aforementioned admonishments, this study, and evidence that exposure of amphibians to low levels of atrazine can increase mortality (Storrs and Kiesecker 2004), impair reproductive development (Hayes et al. 2002a, 2002b, 2003; Tavera-Mendoza et al. 2002a, 2002b), and adversely interact with natural stressors (Boone and James 2003; Kiesecker 2002; Rohr and Palmer 2005), must raise concerns about the role of this widespread, persistent, and mobile herbicide in the international decline of amphibians. Certainly, more research on the effects of atrazine on amphibians is necessary.

In addition to concerns for amphibians, the persistent effects of atrazine should be of general concern because some of the most catastrophic effects of contaminants on wildlife and human populations have been associated with lasting, postexposure effects, such as the enduring effects of organochlorine insecticides [e.g., dichlorodiphenyltrichloroethane (DDT)] and the delayed neurotoxicity of organophosphorus pesticides and various metals (e.g., Hernandez-Avila et al. 1996; Quistad et al. 2003; Rice 1996). Despite these historical cases, studies focusing on carryover effects of contaminants remain surprisingly rare (Jensen and Forbes 2001, Ng and Keough 2003). This is especially disconcerting when one considers that short-term effects are typically inferior to long-term effects for explaining population-level changes and are more likely to give the erroneous impression that certain xenobiotics are innocuous or harmful. Although it can be challenging to quantify long-term effects of stressors across temporal scales (Rohr et al. 2003b), it will likely be necessary to fully understand the impacts of xenobiotics on environmental health.
